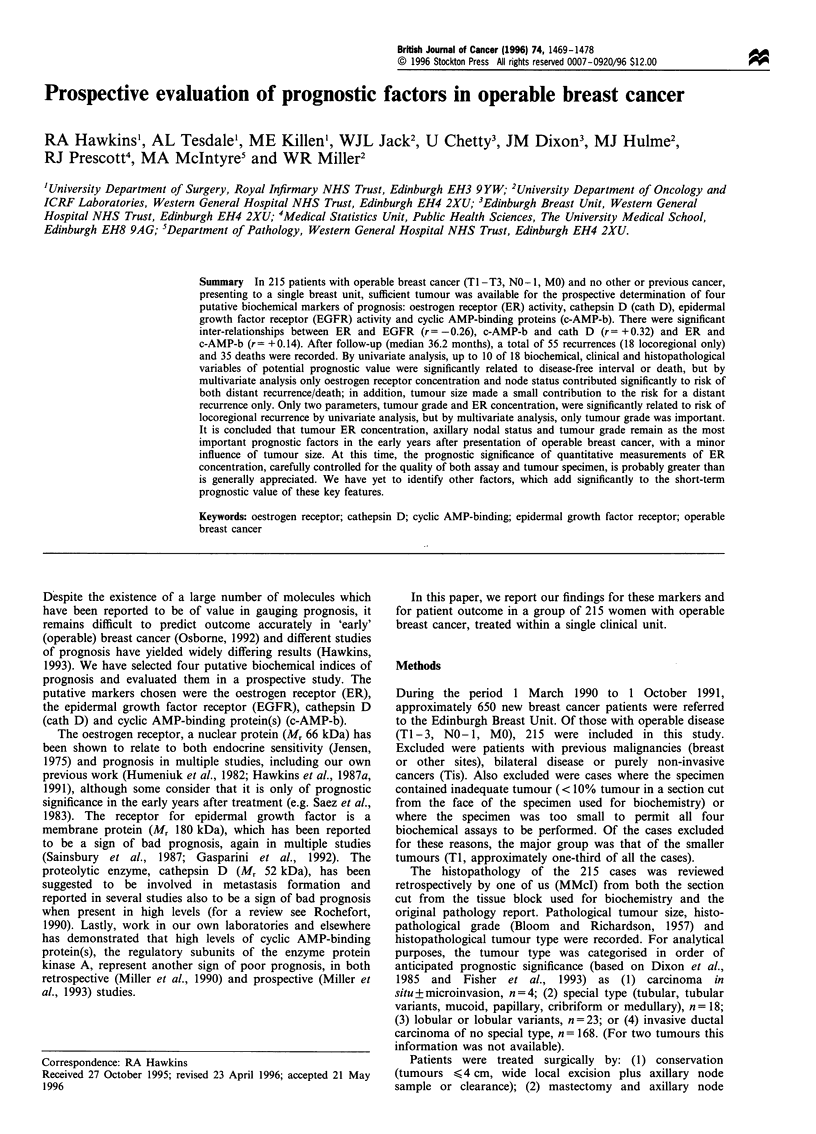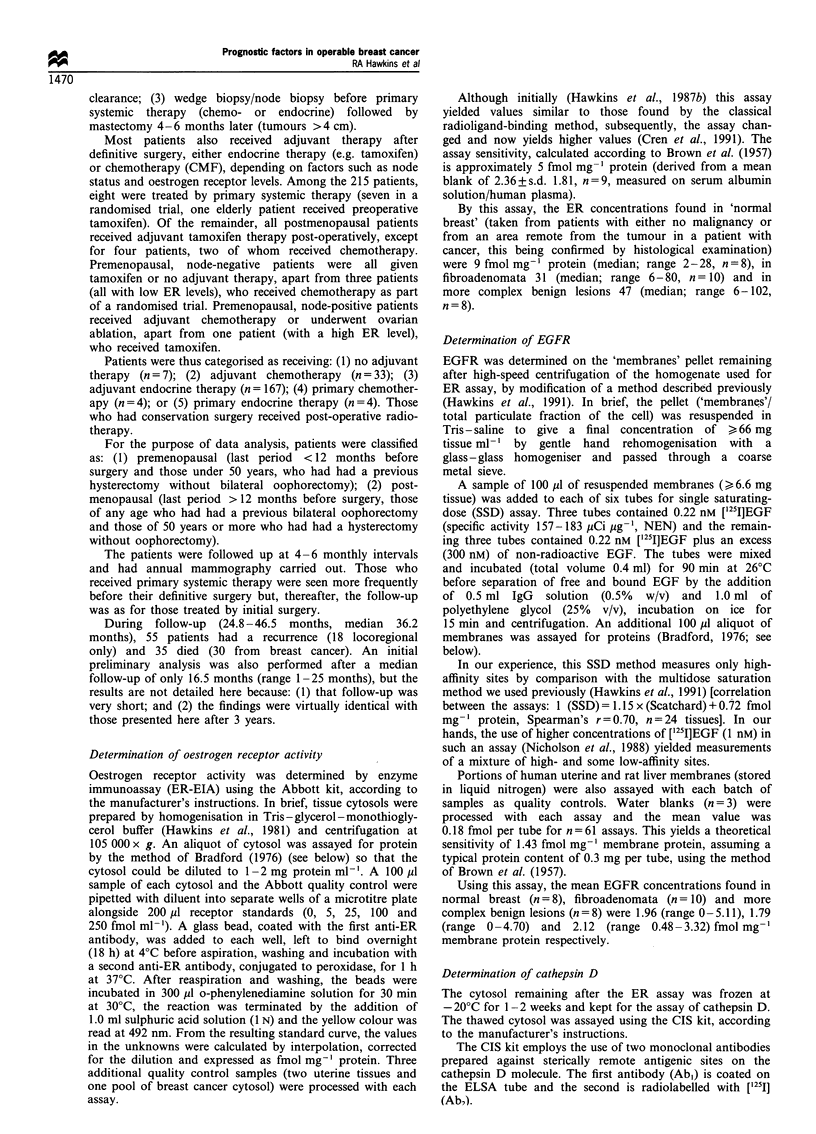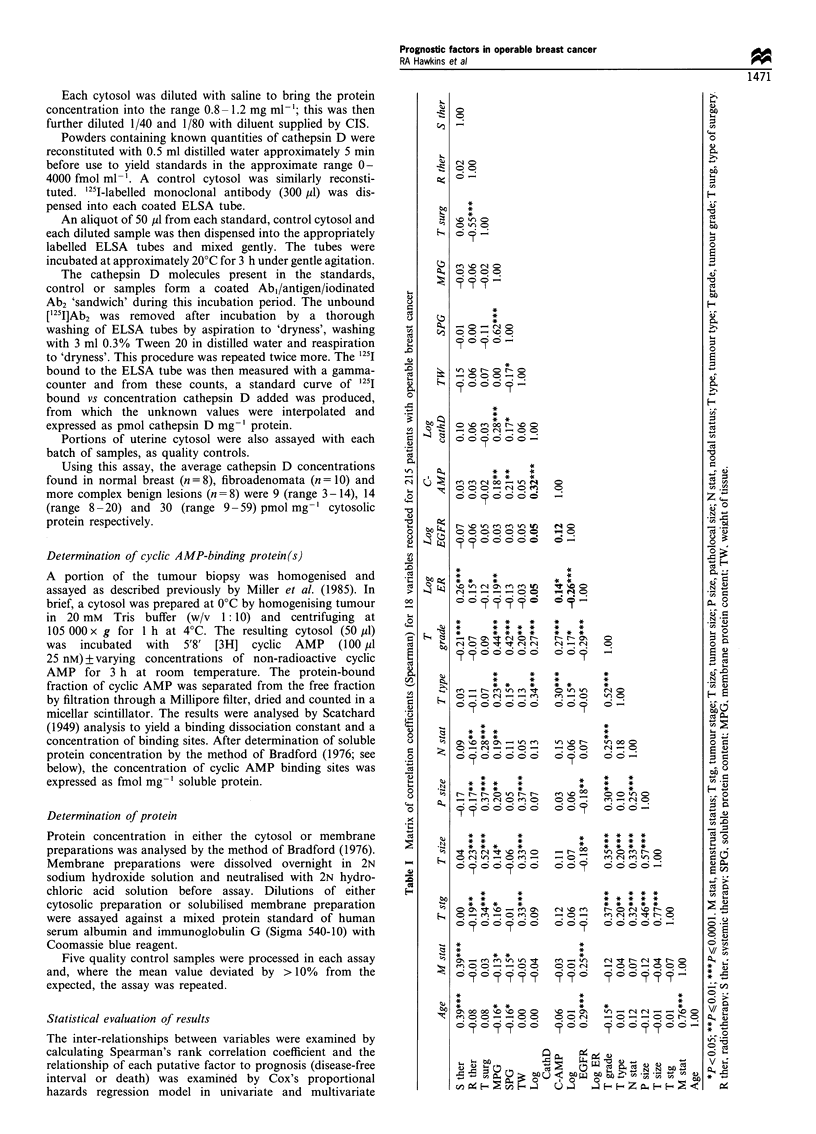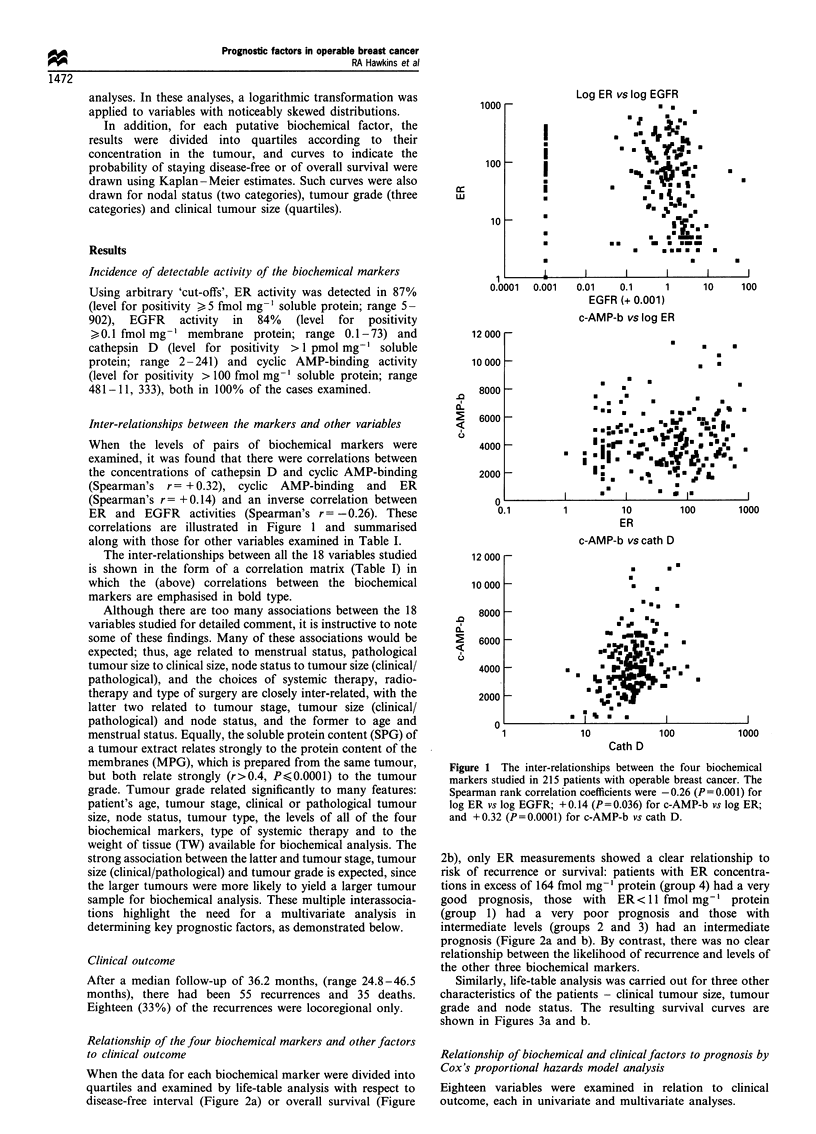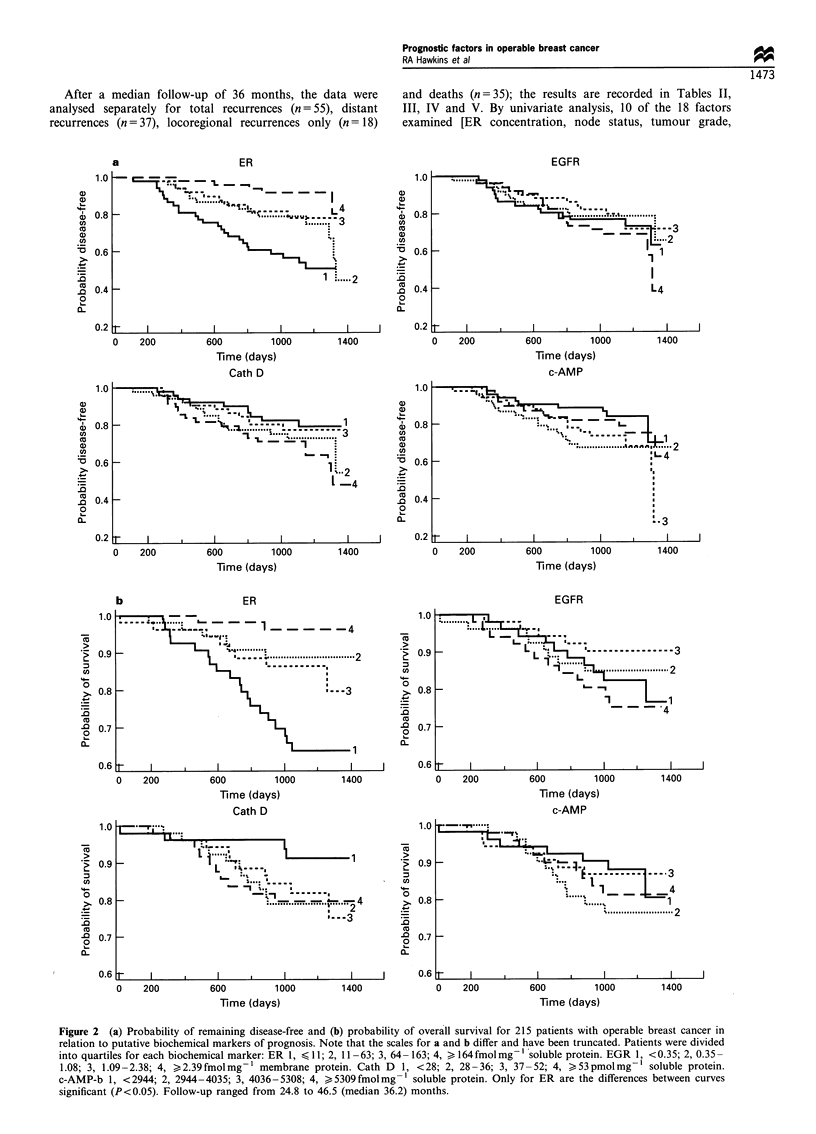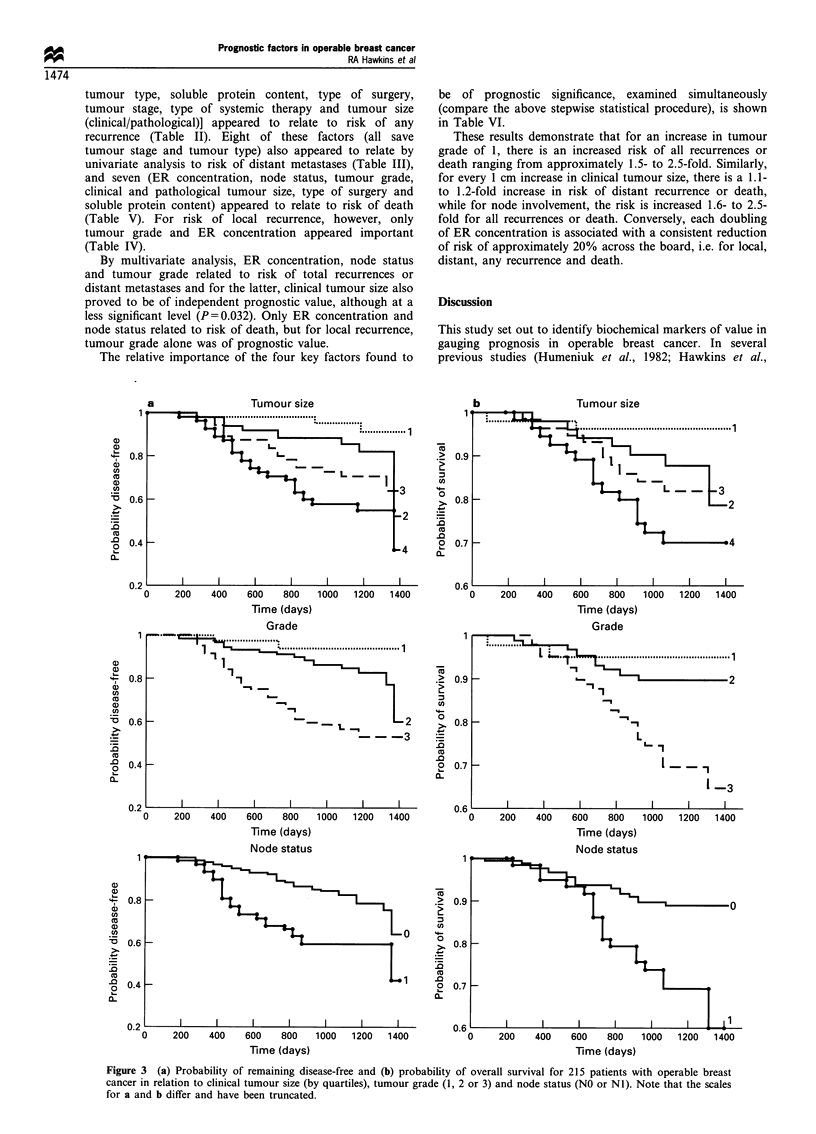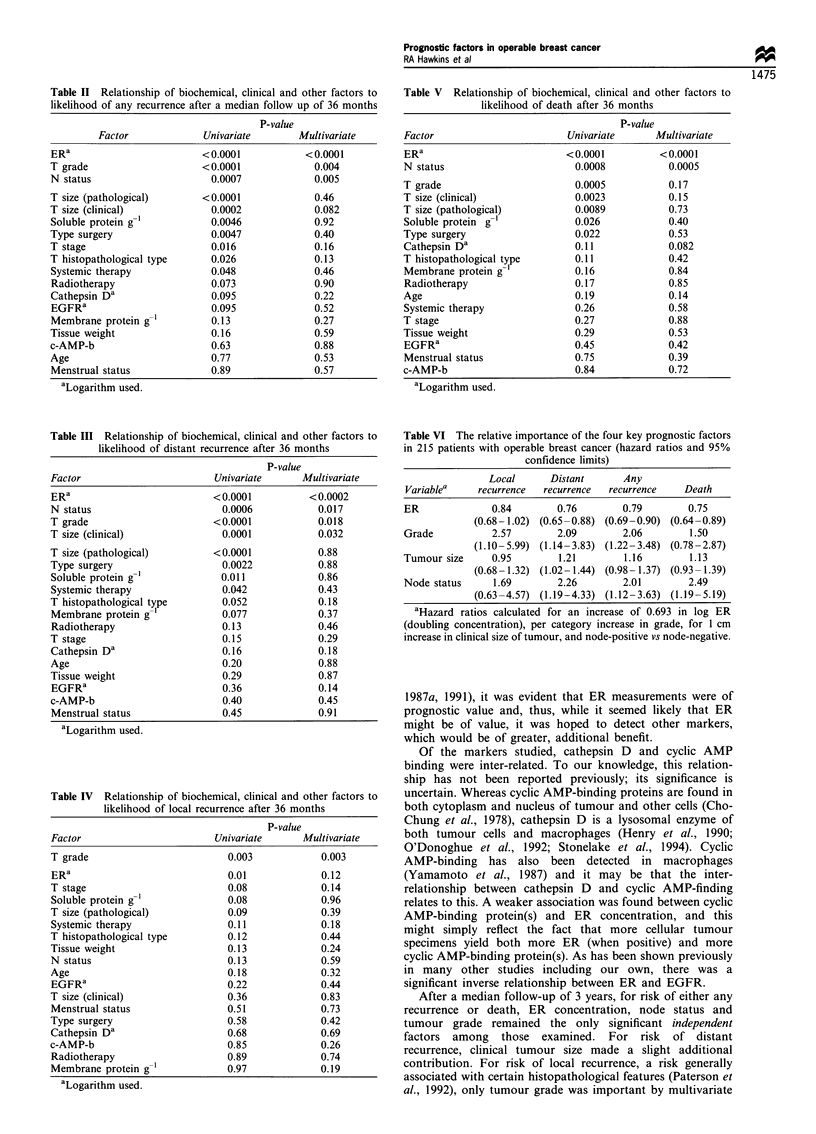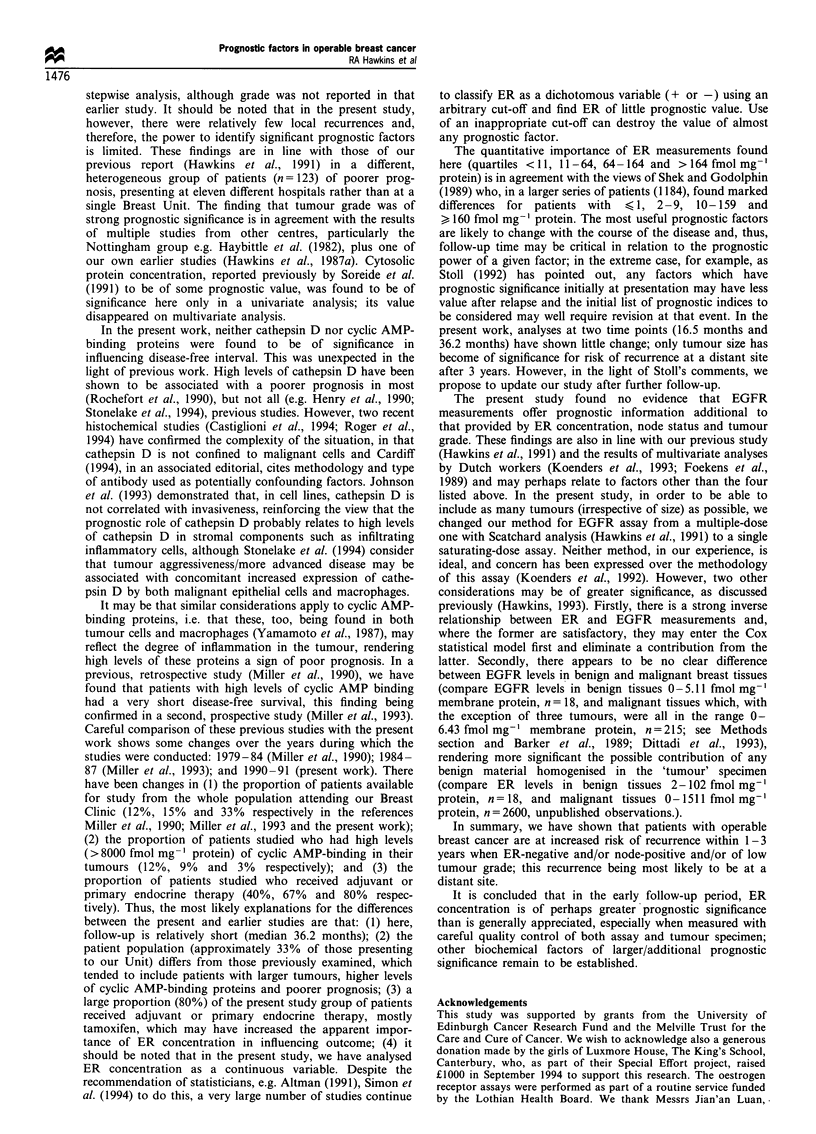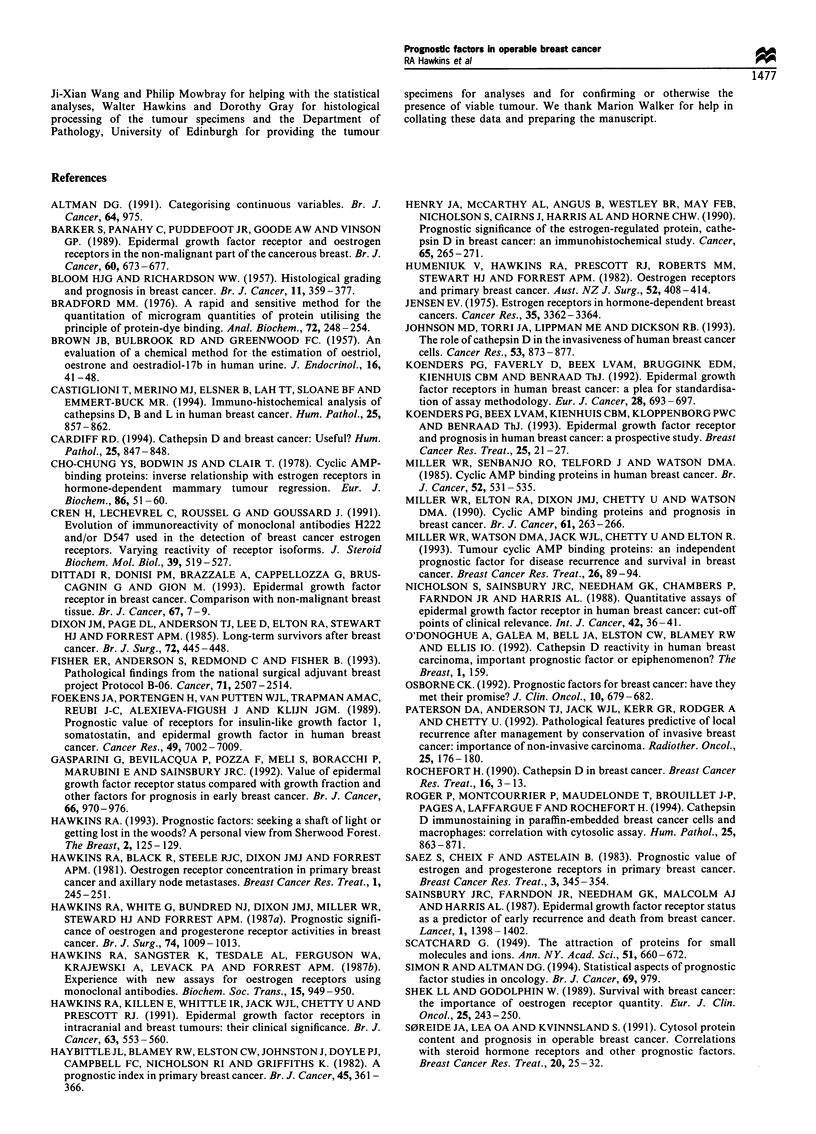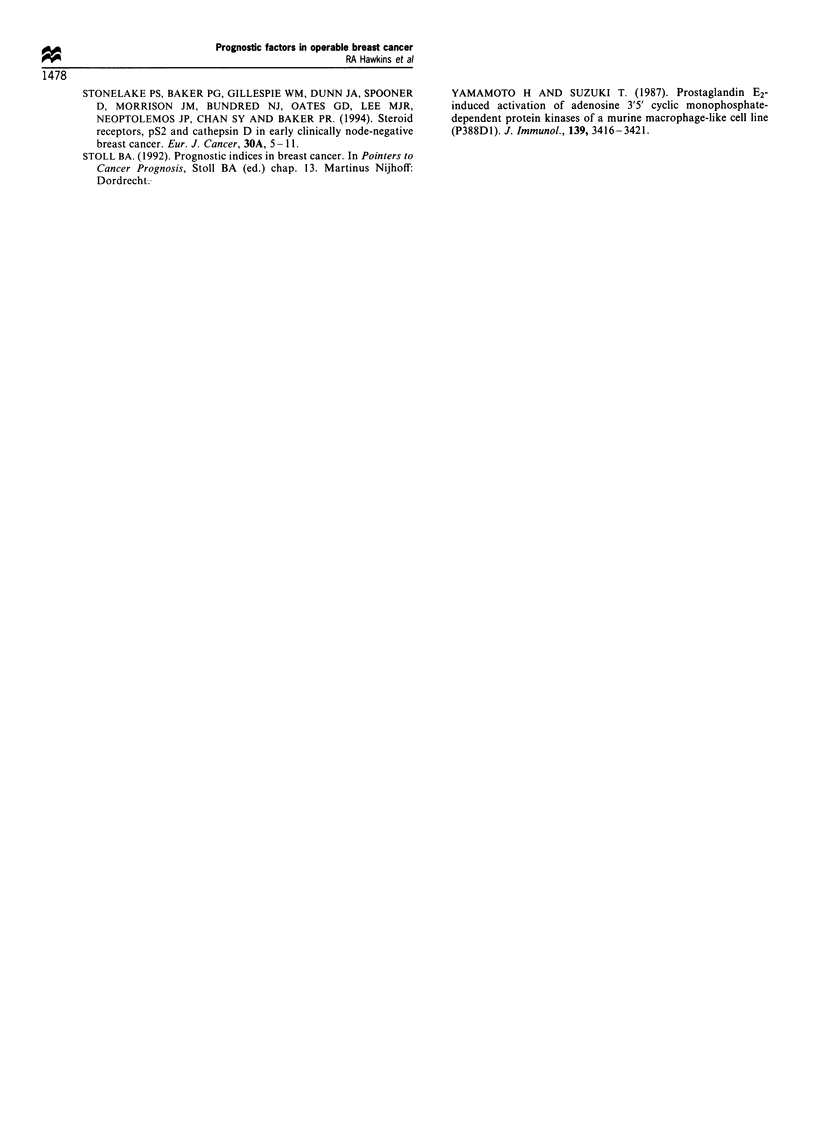# Prospective evaluation of prognostic factors in operable breast cancer.

**DOI:** 10.1038/bjc.1996.567

**Published:** 1996-11

**Authors:** R. A. Hawkins, A. L. Tesdale, M. E. Killen, W. J. Jack, U. Chetty, J. M. Dixon, M. J. Hulme, R. J. Prescott, M. A. McIntyre, W. R. Miller

**Affiliations:** University Department of Surgery, Royal Infirmary NHS Trust, Edinburgh.

## Abstract

In 215 patients with operable breast cancer (T1-T3, N0-1, M0) and no other or previous cancer, presenting to a single breast unit, sufficient tumour was available for the prospective determination of four putative biochemical markers of prognosis: oestrogen receptor (ER) activity, cathepsin D (cath D), epidermal growth factor receptor (EGFR) activity and cyclic AMP-binding proteins (c-AMP-b). There were significant inter-relationships between ER and EGFR (r = -0.26), c-AMP-b and cath D (r = +0.32) and ER and c-AMP-b (r = +0.14). After follow-up (median 36.2 months), a total of 55 recurrences (18 locoregional only) and 35 deaths were recorded. By univariate analysis, up to 10 of 18 biochemical, clinical and histopathological variables of potential prognostic value were significantly related to disease-free interval or death, but by multivariate analysis only oestrogen receptor concentration and node status contributed significantly to risk of both distant recurrence/death; in addition, tumour size made a small contribution to the risk for a distant recurrence only. Only two parameters, tumour grade and ER concentration, were significantly related to risk of locoregional recurrence by univariate analysis, but by multivariate analysis, only tumour grade was important. It is concluded that tumour ER concentration, axillary nodal status and tumour grade remain as the most important prognostic factors in the early years after presentation of operable breast cancer, with a minor influence of tumour size. At this time, the prognostic significance of quantitative measurements of ER concentration, carefully controlled for the quality of both assay and tumour specimen, is probably greater than is generally appreciated. We have yet to identify other factors, which add significantly to the short-term prognostic value of these key features.


British Journal of Cancer (1996) 74, 1469-1478
© 1996 Stockton Press All rights reserved 0007-0920/96 $12.00

### Prospective evaluation of prognostic factors in operable breast cancer

RA Hawkins[1], AL Tesdale[1], ME Killen[1], WJL Jack[2], U Chetty[3], JM Dixon[3], MJ Hulme[2], RJ Prescott[4], MA McIntyre[5] and WR Miller[2]

[1]*University Department of Surgery, Royal Infirmary NHS Trust, Edinburgh EH3 9YW;* [2]*University Department of Oncology and ICRF Laboratories, Western General Hospital NHS Trust, Edinburgh EH4 2XU;* [3]*Edinburgh Breast Unit, Western General Hospital NHS Trust, Edinburgh EH4 2XU;* [4]*Medical Statistics Unit, Public Health Sciences, The University Medical School, Edinburgh EH8 9AG;* [5]*Department of Pathology, Western General Hospital NHS Trust, Edinburgh EH4 2XU.*

**Summary** In 215 patients with operable breast cancer (T1-T3, N0-1, M0) and no other or previous cancer, presenting to a single breast unit, sufficient tumour was available for the prospective determination of four putative biochemical markers of prognosis: oestrogen receptor (ER) activity, cathepsin D (cath D), epidermal growth factor receptor (EGFR) activity and cyclic AMP-binding proteins (c-AMP-b). There were significant inter-relationships between ER and EGFR ($r = -0.26$), c-AMP-b and cath D ($r = +0.32$) and ER and c-AMP-b ($r = +0.14$). After follow-up (median 36.2 months), a total of 55 recurrences (18 locoregional only) and 35 deaths were recorded. By univariate analysis, up to 10 of 18 biochemical, clinical and histopathological variables of potential prognostic value were significantly related to disease-free interval or death, but by multivariate analysis only oestrogen receptor concentration and node status contributed significantly to risk of both distant recurrence/death; in addition, tumour size made a small contribution to the risk for a distant recurrence only. Only two parameters, tumour grade and ER concentration, were significantly related to risk of locoregional recurrence by univariate analysis, but by multivariate analysis, only tumour grade was important. It is concluded that tumour ER concentration, axillary nodal status and tumour grade remain as the most important prognostic factors in the early years after presentation of operable breast cancer, with a minor influence of tumour size. At this time, the prognostic significance of quantitative measurements of ER concentration, carefully controlled for the quality of both assay and tumour specimen, is probably greater than is generally appreciated. We have yet to identify other factors, which add significantly to the short-term prognostic value of these key features.

**Keywords:** oestrogen receptor; cathepsin D; cyclic AMP-binding; epidermal growth factor receptor; operable breast cancer

Despite the existence of a large number of molecules which have been reported to be of value in gauging prognosis, it remains difficult to predict outcome accurately in 'early' (operable) breast cancer (Osborne, 1992) and different studies of prognosis have yielded widely differing results (Hawkins, 1993). We have selected four putative biochemical indices of prognosis and evaluated them in a prospective study. The putative markers chosen were the oestrogen receptor (ER), the epidermal growth factor receptor (EGFR), cathepsin D (cath D) and cyclic AMP-binding protein(s) (c-AMP-b).

The oestrogen receptor, a nuclear protein ($M_r$ 66 kDa) has been shown to relate to both endocrine sensitivity (Jensen, 1975) and prognosis in multiple studies, including our own previous work (Humeniuk *et al.*, 1982; Hawkins *et al.*, 1987*a*, 1991), although some consider that it is only of prognostic significance in the early years after treatment (e.g. Saez *et al.*, 1983). The receptor for epidermal growth factor is a membrane protein ($M_r$ 180 kDa), which has been reported to be a sign of bad prognosis, again in multiple studies (Sainsbury *et al.*, 1987; Gasparini *et al.*, 1992). The proteolytic enzyme, cathepsin D ($M_r$ 52 kDa), has been suggested to be involved in metastasis formation and reported in several studies also to be a sign of bad prognosis when present in high levels (for a review see Rochefort, 1990). Lastly, work in our own laboratories and elsewhere has demonstrated that high levels of cyclic AMP-binding protein(s), the regulatory subunits of the enzyme protein kinase A, represent another sign of poor prognosis, in both retrospective (Miller *et al.*, 1990) and prospective (Miller *et al.*, 1993) studies.

Correspondence: RA Hawkins
Received 27 October 1995; revised 23 April 1996; accepted 21 May 1996

In this paper, we report our findings for these markers and for patient outcome in a group of 215 women with operable breast cancer, treated within a single clinical unit.

### Methods

During the period 1 March 1990 to 1 October 1991, approximately 650 new breast cancer patients were referred to the Edinburgh Breast Unit. Of those with operable disease (T1-3, N0-1, M0), 215 were included in this study. Excluded were patients with previous malignancies (breast or other sites), bilateral disease or purely non-invasive cancers (Tis). Also excluded were cases where the specimen contained inadequate tumour (< 10% tumour in a section cut from the face of the specimen used for biochemistry) or where the specimen was too small to permit all four biochemical assays to be performed. Of the cases excluded for these reasons, the major group was that of the smaller tumours (T1, approximately one-third of all the cases).

The histopathology of the 215 cases was reviewed retrospectively by one of us (MMcI) from both the section cut from the tissue block used for biochemistry and the original pathology report. Pathological tumour size, histopathological grade (Bloom and Richardson, 1957) and histopathological tumour type were recorded. For analytical purposes, the tumour type was categorised in order of anticipated prognostic significance (based on Dixon *et al.*, 1985 and Fisher *et al.*, 1993) as (1) carcinoma *in situ*±microinvasion, $n=4$; (2) special type (tubular, tubular variants, mucoid, papillary, cribriform or medullary), $n=18$; (3) lobular or lobular variants, $n=23$; or (4) invasive ductal carcinoma of no special type, $n=168$. (For two tumours this information was not available).

Patients were treated surgically by: (1) conservation (tumours ⩽4 cm, wide local excision plus axillary node sample or clearance); (2) mastectomy and axillary node clearance; (3) wedge biopsy/node biopsy before primary systemic therapy (chemo- or endocrine) followed by mastectomy 4–6 months later (tumours >4 cm).

Most patients also received adjuvant therapy after definitive surgery, either endocrine therapy (e.g. tamoxifen) or chemotherapy (CMF), depending on factors such as node status and oestrogen receptor levels. Among the 215 patients, eight were treated by primary systemic therapy (seven in a randomised trial, one elderly patient received preoperative tamoxifen). Of the remainder, all postmenopausal patients received adjuvant tamoxifen therapy post-operatively, except for four patients, two of whom received chemotherapy. Premenopausal, node-negative patients were all given tamoxifen or no adjuvant therapy, apart from three patients (all with low ER levels), who received chemotherapy as part of a randomised trial. Premenopausal, node-positive patients received adjuvant chemotherapy or underwent ovarian ablation, apart from one patient (with a high ER level), who received tamoxifen.

Patients were thus categorised as receiving: (1) no adjuvant therapy ($n=7$); (2) adjuvant chemotherapy ($n=33$); (3) adjuvant endocrine therapy ($n=167$); (4) primary chemotherapy ($n=4$); or (5) primary endocrine therapy ($n=4$). Those who had conservation surgery received post-operative radiotherapy.

For the purpose of data analysis, patients were classified as: (1) premenopausal (last period <12 months before surgery and those under 50 years, who had had a previous hysterectomy without bilateral oophorectomy); (2) postmenopausal (last period >12 months before surgery, those of any age who had had a previous bilateral oophorectomy and those of 50 years or more who had had a hysterectomy without oophorectomy).

The patients were followed up at 4–6 monthly intervals and had annual mammography carried out. Those who received primary systemic therapy were seen more frequently before their definitive surgery but, thereafter, the follow-up was as for those treated by initial surgery.

During follow-up (24.8–46.5 months, median 36.2 months), 55 patients had a recurrence (18 locoregional only) and 35 died (30 from breast cancer). An initial preliminary analysis was also performed after a median follow-up of only 16.5 months (range 1–25 months), but the results are not detailed here because: (1) that follow-up was very short; and (2) the findings were virtually identical with those presented here after 3 years.

*Determination of oestrogen receptor activity*

Oestrogen receptor activity was determined by enzyme immunoassay (ER-EIA) using the Abbott kit, according to the manufacturer's instructions. In brief, tissue cytosols were prepared by homogenisation in Tris–glycerol–monothioglycerol buffer (Hawkins *et al.*, 1981) and centrifugation at 105 000 × *g*. An aliquot of cytosol was assayed for protein by the method of Bradford (1976) (see below) so that the cytosol could be diluted to 1–2 mg protein $ml^{-1}$. A 100 μl sample of each cytosol and the Abbott quality control were pipetted with diluent into separate wells of a microtitre plate alongside 200 μl receptor standards (0, 5, 25, 100 and 250 fmol $ml^{-1}$). A glass bead, coated with the first anti-ER antibody, was added to each well, left to bind overnight (18 h) at 4°C before aspiration, washing and incubation with a second anti-ER antibody, conjugated to peroxidase, for 1 h at 37°C. After reaspiration and washing, the beads were incubated in 300 μl o-phenylenediamine solution for 30 min at 30°C, the reaction was terminated by the addition of 1.0 ml sulphuric acid solution (1 N) and the yellow colour was read at 492 nm. From the resulting standard curve, the values in the unknowns were calculated by interpolation, corrected for the dilution and expressed as fmol $mg^{-1}$ protein. Three additional quality control samples (two uterine tissues and one pool of breast cancer cytosol) were processed with each assay.

Although initially (Hawkins *et al.*, 1987*b*) this assay yielded values similar to those found by the classical radioligand-binding method, subsequently, the assay changed and now yields higher values (Cren *et al.*, 1991). The assay sensitivity, calculated according to Brown *et al.* (1957) is approximately 5 fmol $mg^{-1}$ protein (derived from a mean blank of 2.36 ± s.d. 1.81, $n=9$, measured on serum albumin solution/human plasma).

By this assay, the ER concentrations found in 'normal breast' (taken from patients with either no malignancy or from an area remote from the tumour in a patient with cancer, this being confirmed by histological examination) were 9 fmol $mg^{-1}$ protein (median; range 2–28, $n=8$), in fibroadenomata 31 (median; range 6–80, $n=10$) and in more complex benign lesions 47 (median; range 6–102, $n=8$).

*Determination of EGFR*

EGFR was determined on the 'membranes' pellet remaining after high-speed centrifugation of the homogenate used for ER assay, by modification of a method described previously (Hawkins *et al.*, 1991). In brief, the pellet ('membranes'/total particulate fraction of the cell) was resuspended in Tris–saline to give a final concentration of ⩾66 mg tissue $ml^{-1}$ by gentle hand rehomogenisation with a glass–glass homogeniser and passed through a coarse metal sieve.

A sample of 100 μl of resuspended membranes (⩾6.6 mg tissue) was added to each of six tubes for single saturating-dose (SSD) assay. Three tubes contained 0.22 nM [$^{125}$I]EGF (specific activity 157–183 μCi $\mu g^{-1}$, NEN) and the remaining three tubes contained 0.22 nM [$^{125}$I]EGF plus an excess (300 nM) of non-radioactive EGF. The tubes were mixed and incubated (total volume 0.4 ml) for 90 min at 26°C before separation of free and bound EGF by the addition of 0.5 ml IgG solution (0.5% w/v) and 1.0 ml of polyethylene glycol (25% v/v), incubation on ice for 15 min and centrifugation. An additional 100 μl aliquot of membranes was assayed for proteins (Bradford, 1976; see below).

In our experience, this SSD method measures only high-affinity sites by comparison with the multidose saturation method we used previously (Hawkins *et al.*, 1991) [correlation between the assays: 1 (SSD) = 1.15 × (Scatchard) + 0.72 fmol $mg^{-1}$ protein, Spearman's $r=0.70$, $n=24$ tissues]. In our hands, the use of higher concentrations of [$^{125}$I]EGF (1 nM) in such an assay (Nicholson *et al.*, 1988) yielded measurements of a mixture of high- and some low-affinity sites.

Portions of human uterine and rat liver membranes (stored in liquid nitrogen) were also assayed with each batch of samples as quality controls. Water blanks ($n=3$) were processed with each assay and the mean value was 0.18 fmol per tube for $n=61$ assays. This yields a theoretical sensitivity of 1.43 fmol $mg^{-1}$ membrane protein, assuming a typical protein content of 0.3 mg per tube, using the method of Brown *et al.* (1957).

Using this assay, the mean EGFR concentrations found in normal breast ($n=8$), fibroadenomata ($n=10$) and more complex benign lesions ($n=8$) were 1.96 (range 0–5.11), 1.79 (range 0–4.70) and 2.12 (range 0.48–3.32) fmol $mg^{-1}$ membrane protein respectively.

*Determination of cathepsin D*

The cytosol remaining after the ER assay was frozen at −20°C for 1–2 weeks and kept for the assay of cathepsin D. The thawed cytosol was assayed using the CIS kit, according to the manufacturer's instructions.

The CIS kit employs the use of two monoclonal antibodies prepared against sterically remote antigenic sites on the cathepsin D molecule. The first antibody ($Ab_1$) is coated on the ELSA tube and the second is radiolabelled with [$^{125}$I] ($Ab_2$).

Each cytosol was diluted with saline to bring the protein concentration into the range 0.8–1.2 mg ml$^{-1}$; this was then further diluted 1/40 and 1/80 with diluent supplied by CIS.

Powders containing known quantities of cathepsin D were reconstituted with 0.5 ml distilled water approximately 5 min before use to yield standards in the approximate range 0–4000 fmol ml$^{-1}$. A control cytosol was similarly reconstituted. $^{125}$I-labelled monoclonal antibody (300 $\mu$l) was dispensed into each coated ELSA tube.

An aliquot of 50 $\mu$l from each standard, control cytosol and each diluted sample was then dispensed into the appropriately labelled ELSA tubes and mixed gently. The tubes were incubated at approximately 20°C for 3 h under gentle agitation.

The cathepsin D molecules present in the standards, control or samples form a coated $Ab_1$/antigen/iodinated $Ab_2$ 'sandwich' during this incubation period. The unbound [$^{125}$I]$Ab_2$ was removed after incubation by a thorough washing of ELSA tubes by aspiration to 'dryness', washing with 3 ml 0.3% Tween 20 in distilled water and reaspiration to 'dryness'. This procedure was repeated twice more. The $^{125}$I bound to the ELSA tube was then measured with a gamma-counter and from these counts, a standard curve of $^{125}$I bound *vs* concentration cathepsin D added was produced, from which the unknown values were interpolated and expressed as pmol cathepsin D mg$^{-1}$ protein.

Portions of uterine cytosol were also assayed with each batch of samples, as quality controls.

Using this assay, the average cathepsin D concentrations found in normal breast ($n=8$), fibroadenomata ($n=10$) and more complex benign lesions ($n=8$) were 9 (range 3–14), 14 (range 8–20) and 30 (range 9–59) pmol mg$^{-1}$ cytosolic protein respectively.

*Determination of cyclic AMP-binding protein(s)*

A portion of the tumour biopsy was homogenised and assayed as described previously by Miller *et al.* (1985). In brief, a cytosol was prepared at 0°C by homogenising tumour in 20 mM Tris buffer (w/v 1:10) and centrifuging at 105 000 × *g* for 1 h at 4°C. The resulting cytosol (50 $\mu$l) was incubated with 5′8′ [3H] cyclic AMP (100 $\mu$l 25 nM) ± varying concentrations of non-radioactive cyclic AMP for 3 h at room temperature. The protein-bound fraction of cyclic AMP was separated from the free fraction by filtration through a Millipore filter, dried and counted in a micellar scintillator. The results were analysed by Scatchard (1949) analysis to yield a binding dissociation constant and a concentration of binding sites. After determination of soluble protein concentration by the method of Bradford (1976; see below), the concentration of cyclic AMP binding sites was expressed as fmol mg$^{-1}$ soluble protein.

*Determination of protein*

Protein concentration in either the cytosol or membrane preparations was analysed by the method of Bradford (1976). Membrane preparations were dissolved overnight in 2N sodium hydroxide solution and neutralised with 2N hydrochloric acid solution before assay. Dilutions of either cytosolic preparation or solubilised membrane preparation were assayed against a mixed protein standard of human serum albumin and immunoglobulin G (Sigma 540-10) with Coomassie blue reagent.

Five quality control samples were processed in each assay and, where the mean value deviated by >10% from the expected, the assay was repeated.

*Statistical evaluation of results*

The inter-relationships between variables were examined by calculating Spearman's rank correlation coefficient and the relationship of each putative factor to prognosis (disease-free interval or death) was examined by Cox's proportional hazards regression model in univariate and multivariate

**Table I** Matrix of correlation coefficients (Spearman) for 18 variables recorded for 215 patients with operable breast cancer

| | *Age* | *M stat* | *T stg* | *T size* | *P size* | *N stat* | *T type* | *T grade* | *Log ER* | *Log EGFR* | *C-AMP* | *Log cathD* | *TW* | *SPG* | *MPG* | *T surg* | *R ther* | *S ther* |
|---|---|---|---|---|---|---|---|---|---|---|---|---|---|---|---|---|---|---|
| S ther | 0.39*** | 0.39*** | 0.00 | 0.04 | –0.17 | 0.09 | 0.03 | –0.21*** | 0.26*** | –0.07 | 0.03 | 0.10 | –0.15 | –0.01 | –0.03 | 0.06 | 0.02 | 1.00 |
| R ther | –0.08 | –0.01 | –0.19** | –0.23*** | –0.17** | –0.16** | –0.11 | –0.07 | 0.15* | –0.06 | 0.03 | 0.06 | 0.06 | 0.00 | –0.06 | –0.55*** | 1.00 | |
| T surg | 0.08 | 0.03 | 0.34*** | 0.52*** | 0.37*** | 0.28*** | 0.07 | 0.09 | –0.12 | 0.05 | –0.02 | –0.03 | 0.07 | –0.11 | –0.02 | 1.00 | | |
| MPG | –0.16* | –0.13* | 0.16* | 0.14* | 0.20** | 0.19** | 0.23*** | 0.44*** | –0.19** | 0.03 | 0.18** | 0.28*** | 0.00 | 0.62*** | 1.00 | | | |
| SPG | –0.16* | –0.15* | –0.01 | –0.06 | 0.05 | 0.11 | 0.15* | 0.42*** | –0.13 | 0.03 | 0.21** | 0.17* | –0.17* | 1.00 | | | | |
| TW | 0.00 | –0.05 | 0.33*** | 0.33*** | 0.37*** | 0.05 | 0.13 | 0.20** | –0.03 | 0.05 | 0.05 | 0.06 | 1.00 | | | | | |
| Log CathD | 0.00 | –0.04 | 0.09 | 0.10 | 0.07 | 0.13 | 0.34*** | 0.27*** | **0.05** | **0.05** | **0.32***** | 1.00 | | | | | | |
| C-AMP | –0.06 | –0.03 | 0.12 | 0.11 | 0.03 | 0.15 | 0.30*** | 0.27*** | **0.14*** | **0.12** | 1.00 | | | | | | | |
| Log | 0.01 | –0.01 | 0.06 | 0.07 | 0.06 | –0.06 | 0.15* | 0.17* | **–0.26***** | 1.00 | | | | | | | | |
| EGFR | 0.29*** | 0.25*** | –0.13 | –0.18** | –0.18** | 0.07 | –0.05 | –0.29*** | 1.00 | | | | | | | | | |
| Log ER | | | | | | | | | | | | | | | | | | |
| T grade | –0.15* | –0.12 | 0.37*** | 0.35*** | 0.30*** | 0.25*** | 0.52*** | 1.00 | | | | | | | | | | |
| T type | 0.01 | 0.04 | 0.20** | 0.20*** | 0.10 | 0.18 | 1.00 | | | | | | | | | | | |
| N stat | 0.12 | 0.07 | 0.32*** | 0.33*** | 0.25*** | 1.00 | | | | | | | | | | | | |
| P size | –0.12 | –0.12 | 0.46*** | 0.57*** | 1.00 | | | | | | | | | | | | | |
| T size | –0.01 | –0.04 | 0.77*** | 1.00 | | | | | | | | | | | | | | |
| T stg | 0.01 | –0.07 | 1.00 | | | | | | | | | | | | | | | |
| M stat | 0.76*** | 1.00 | | | | | | | | | | | | | | | | |
| Age | 1.00 | | | | | | | | | | | | | | | | | |

*$P<0.05$; **$P\leqslant0.01$; ***$P\leqslant0.0001$. M stat, menstrual status; T stg, tumour stage; T size, tumour size; P size, patholocal size; N stat, nodal status; T type, tumour type; T grade, tumour grade; T surg, type of surgery. R ther, radiotherapy; S ther, systemic therapy; SPG, soluble protein content; MPG, membrane protein content; TW, weight of tissue.

analyses. In these analyses, a logarithmic transformation was applied to variables with noticeably skewed distributions.

In addition, for each putative biochemical factor, the results were divided into quartiles according to their concentration in the tumour, and curves to indicate the probability of staying disease-free or of overall survival were drawn using Kaplan–Meier estimates. Such curves were also drawn for nodal status (two categories), tumour grade (three categories) and clinical tumour size (quartiles).

### Results

*Incidence of detectable activity of the biochemical markers*

Using arbitrary 'cut-offs', ER activity was detected in 87% (level for positivity $\geqslant 5$ fmol $mg^{-1}$ soluble protein; range 5–902), EGFR activity in 84% (level for positivity $\geqslant 0.1$ fmol $mg^{-1}$ membrane protein; range 0.1–73) and cathepsin D (level for positivity $>1$ pmol $mg^{-1}$ soluble protein; range 2–241) and cyclic AMP-binding activity (level for positivity $>100$ fmol $mg^{-1}$ soluble protein; range 481–11, 333), both in 100% of the cases examined.

*Inter-relationships between the markers and other variables*

When the levels of pairs of biochemical markers were examined, it was found that there were correlations between the concentrations of cathepsin D and cyclic AMP-binding (Spearman's $r = +0.32$), cyclic AMP-binding and ER (Spearman's $r = +0.14$) and an inverse correlation between ER and EGFR activities (Spearman's $r = -0.26$). These correlations are illustrated in Figure 1 and summarised along with those for other variables examined in Table I.

The inter-relationships between all the 18 variables studied is shown in the form of a correlation matrix (Table I) in which the (above) correlations between the biochemical markers are emphasised in bold type.

Although there are too many associations between the 18 variables studied for detailed comment, it is instructive to note some of these findings. Many of these associations would be expected; thus, age related to menstrual status, pathological tumour size to clinical size, node status to tumour size (clinical/pathological), and the choices of systemic therapy, radiotherapy and type of surgery are closely inter-related, with the latter two related to tumour stage, tumour size (clinical/pathological) and node status, and the former to age and menstrual status. Equally, the soluble protein content (SPG) of a tumour extract relates strongly to the protein content of the membranes (MPG), which is prepared from the same tumour, but both relate strongly ($r > 0.4$, $P \leqslant 0.0001$) to the tumour grade. Tumour grade related significantly to many features: patient's age, tumour stage, clinical or pathological tumour size, node status, tumour type, the levels of all of the four biochemical markers, type of systemic therapy and to the weight of tissue (TW) available for biochemical analysis. The strong association between the latter and tumour stage, tumour size (clinical/pathological) and tumour grade is expected, since the larger tumours were more likely to yield a larger tumour sample for biochemical analysis. These multiple interassociations highlight the need for a multivariate analysis in determining key prognostic factors, as demonstrated below.

*Clinical outcome*

After a median follow-up of 36.2 months, (range 24.8–46.5 months), there had been 55 recurrences and 35 deaths. Eighteen (33%) of the recurrences were locoregional only.

*Relationship of the four biochemical markers and other factors to clinical outcome*

When the data for each biochemical marker were divided into quartiles and examined by life-table analysis with respect to disease-free interval (Figure 2a) or overall survival (Figure 2b), only ER measurements showed a clear relationship to risk of recurrence or survival: patients with ER concentrations in excess of 164 fmol $mg^{-1}$ protein (group 4) had a very good prognosis, those with ER<11 fmol $mg^{-1}$ protein (group 1) had a very poor prognosis and those with intermediate levels (groups 2 and 3) had an intermediate prognosis (Figure 2a and b). By contrast, there was no clear relationship between the likelihood of recurrence and levels of the other three biochemical markers.

**Figure 1** The inter-relationships between the four biochemical markers studied in 215 patients with operable breast cancer. The Spearman rank correlation coefficients were $-0.26$ ($P = 0.001$) for log ER *vs* log EGFR; $+0.14$ ($P = 0.036$) for c-AMP-b *vs* log ER; and $+0.32$ ($P = 0.0001$) for c-AMP-b *vs* cath D.

Similarly, life-table analysis was carried out for three other characteristics of the patients – clinical tumour size, tumour grade and node status. The resulting survival curves are shown in Figures 3a and b.

*Relationship of biochemical and clinical factors to prognosis by Cox's proportional hazards model analysis*

Eighteen variables were examined in relation to clinical outcome, each in univariate and multivariate analyses.

After a median follow-up of 36 months, the data were analysed separately for total recurrences ($n=55$), distant recurrences ($n=37$), locoregional recurrences only ($n=18$) and deaths ($n=35$); the results are recorded in Tables II, III, IV and V. By univariate analysis, 10 of the 18 factors examined [ER concentration, node status, tumour grade,

**Figure 2** (**a**) Probability of remaining disease-free and (**b**) probability of overall survival for 215 patients with operable breast cancer in relation to putative biochemical markers of prognosis. Note that the scales for **a** and **b** differ and have been truncated. Patients were divided into quartiles for each biochemical marker: ER 1, ⩽11; 2, 11–63; 3, 64–163; 4, ⩾164 fmol mg$^{-1}$ soluble protein. EGR 1, <0.35; 2, 0.35–1.08; 3, 1.09–2.38; 4, ⩾2.39 fmol mg$^{-1}$ membrane protein. Cath D 1, <28; 2, 28–36; 3, 37–52; 4, ⩾53 pmol mg$^{-1}$ soluble protein. c-AMP-b 1, <2944; 2, 2944–4035; 3, 4036–5308; 4, ⩾5309 fmol mg$^{-1}$ soluble protein. Only for ER are the differences between curves significant ($P<0.05$). Follow-up ranged from 24.8 to 46.5 (median 36.2) months.

tumour type, soluble protein content, type of surgery, tumour stage, type of systemic therapy and tumour size (clinical/pathological)] appeared to relate to risk of any recurrence (Table II). Eight of these factors (all save tumour stage and tumour type) also appeared to relate by univariate analysis to risk of distant metastases (Table III), and seven (ER concentration, node status, tumour grade, clinical and pathological tumour size, type of surgery and soluble protein content) appeared to relate to risk of death (Table V). For risk of local recurrence, however, only tumour grade and ER concentration appeared important (Table IV).

By multivariate analysis, ER concentration, node status and tumour grade related to risk of total recurrences or distant metastases and for the latter, clinical tumour size also proved to be of independent prognostic value, although at a less significant level ($P=0.032$). Only ER concentration and node status related to risk of death, but for local recurrence, tumour grade alone was of prognostic value.

The relative importance of the four key factors found to be of prognostic significance, examined simultaneously (compare the above stepwise statistical procedure), is shown in Table VI.

These results demonstrate that for an increase in tumour grade of 1, there is an increased risk of all recurrences or death ranging from approximately 1.5- to 2.5-fold. Similarly, for every 1 cm increase in clinical tumour size, there is a 1.1- to 1.2-fold increase in risk of distant recurrence or death, while for node involvement, the risk is increased 1.6- to 2.5-fold for all recurrences or death. Conversely, each doubling of ER concentration is associated with a consistent reduction of risk of approximately 20% across the board, i.e. for local, distant, any recurrence and death.

### Discussion

This study set out to identify biochemical markers of value in gauging prognosis in operable breast cancer. In several previous studies (Humeniuk *et al.*, 1982; Hawkins *et al.*,

**Figure 3** (**a**) Probability of remaining disease-free and (**b**) probability of overall survival for 215 patients with operable breast cancer in relation to clinical tumour size (by quartiles), tumour grade (1, 2 or 3) and node status (N0 or N1). Note that the scales for **a** and **b** differ and have been truncated.

**Table II** Relationship of biochemical, clinical and other factors to likelihood of any recurrence after a median follow up of 36 months

| Factor | P-value Univariate | P-value Multivariate |
|---|---|---|
| ER[a] | <0.0001 | <0.0001 |
| T grade | <0.0001 | 0.004 |
| N status | 0.0007 | 0.005 |
| T size (pathological) | <0.0001 | 0.46 |
| T size (clinical) | 0.0002 | 0.082 |
| Soluble protein $g^{-1}$ | 0.0046 | 0.92 |
| Type surgery | 0.0047 | 0.40 |
| T stage | 0.016 | 0.16 |
| T histopathological type | 0.026 | 0.13 |
| Systemic therapy | 0.048 | 0.46 |
| Radiotherapy | 0.073 | 0.90 |
| Cathepsin D[a] | 0.095 | 0.22 |
| EGFR[a] | 0.095 | 0.52 |
| Membrane protein $g^{-1}$ | 0.13 | 0.27 |
| Tissue weight | 0.16 | 0.59 |
| c-AMP-b | 0.63 | 0.88 |
| Age | 0.77 | 0.53 |
| Menstrual status | 0.89 | 0.57 |

[a]Logarithm used.

**Table III** Relationship of biochemical, clinical and other factors to likelihood of distant recurrence after 36 months

| Factor | P-value Univariate | P-value Multivariate |
|---|---|---|
| ER[a] | <0.0001 | <0.0002 |
| N status | 0.0006 | 0.017 |
| T grade | <0.0001 | 0.018 |
| T size (clinical) | 0.0001 | 0.032 |
| T size (pathological) | <0.0001 | 0.88 |
| Type surgery | 0.0022 | 0.88 |
| Soluble protein $g^{-1}$ | 0.011 | 0.86 |
| Systemic therapy | 0.042 | 0.43 |
| T histopathological type | 0.052 | 0.18 |
| Membrane protein $g^{-1}$ | 0.077 | 0.37 |
| Radiotherapy | 0.13 | 0.46 |
| T stage | 0.15 | 0.29 |
| Cathepsin D[a] | 0.16 | 0.18 |
| Age | 0.20 | 0.88 |
| Tissue weight | 0.29 | 0.87 |
| EGFR[a] | 0.36 | 0.14 |
| c-AMP-b | 0.40 | 0.45 |
| Menstrual status | 0.45 | 0.91 |

[a]Logarithm used.

**Table IV** Relationship of biochemical, clinical and other factors to likelihood of local recurrence after 36 months

| Factor | P-value Univariate | P-value Multivariate |
|---|---|---|
| T grade | 0.003 | 0.003 |
| ER[a] | 0.01 | 0.12 |
| T stage | 0.08 | 0.14 |
| Soluble protein $g^{-1}$ | 0.08 | 0.96 |
| T size (pathological) | 0.09 | 0.39 |
| Systemic therapy | 0.11 | 0.18 |
| T histopathological type | 0.12 | 0.44 |
| Tissue weight | 0.13 | 0.24 |
| N status | 0.13 | 0.59 |
| Age | 0.18 | 0.32 |
| EGFR[a] | 0.22 | 0.44 |
| T size (clinical) | 0.36 | 0.83 |
| Menstrual status | 0.51 | 0.73 |
| Type surgery | 0.58 | 0.42 |
| Cathepsin D[a] | 0.68 | 0.69 |
| c-AMP-b | 0.85 | 0.26 |
| Radiotherapy | 0.89 | 0.74 |
| Membrane protein $g^{-1}$ | 0.97 | 0.19 |

[a]Logarithm used.

**Table V** Relationship of biochemical, clinical and other factors to likelihood of death after 36 months

| Factor | P-value Univariate | P-value Multivariate |
|---|---|---|
| ER[a] | <0.0001 | <0.0001 |
| N status | 0.0008 | 0.0005 |
| T grade | 0.0005 | 0.17 |
| T size (clinical) | 0.0023 | 0.15 |
| T size (pathological) | 0.0089 | 0.73 |
| Soluble protein $g^{-1}$ | 0.026 | 0.40 |
| Type surgery | 0.022 | 0.53 |
| Cathepsin D[a] | 0.11 | 0.082 |
| T histopathological type | 0.11 | 0.42 |
| Membrane protein $g^{-1}$ | 0.16 | 0.84 |
| Radiotherapy | 0.17 | 0.85 |
| Age | 0.19 | 0.14 |
| Systemic therapy | 0.26 | 0.58 |
| T stage | 0.27 | 0.88 |
| Tissue weight | 0.29 | 0.53 |
| EGFR[a] | 0.45 | 0.42 |
| Menstrual status | 0.75 | 0.39 |
| c-AMP-b | 0.84 | 0.72 |

[a]Logarithm used.

**Table VI** The relative importance of the four key prognostic factors in 215 patients with operable breast cancer (hazard ratios and 95% confidence limits)

| Variable[a] | Local recurrence | Distant recurrence | Any recurrence | Death |
|---|---|---|---|---|
| ER | 0.84 | 0.76 | 0.79 | 0.75 |
| | (0.68-1.02) | (0.65-0.88) | (0.69-0.90) | (0.64-0.89) |
| Grade | 2.57 | 2.09 | 2.06 | 1.50 |
| | (1.10-5.99) | (1.14-3.83) | (1.22-3.48) | (0.78-2.87) |
| Tumour size | 0.95 | 1.21 | 1.16 | 1.13 |
| | (0.68-1.32) | (1.02-1.44) | (0.98-1.37) | (0.93-1.39) |
| Node status | 1.69 | 2.26 | 2.01 | 2.49 |
| | (0.63-4.57) | (1.19-4.33) | (1.12-3.63) | (1.19-5.19) |

[a]Hazard ratios calculated for an increase of 0.693 in log ER (doubling concentration), per category increase in grade, for 1 cm increase in clinical size of tumour, and node-positive *vs* node-negative.

1987*a*, 1991), it was evident that ER measurements were of prognostic value and, thus, while it seemed likely that ER might be of value, it was hoped to detect other markers, which would be of greater, additional benefit.

Of the markers studied, cathepsin D and cyclic AMP binding were inter-related. To our knowledge, this relationship has not been reported previously; its significance is uncertain. Whereas cyclic AMP-binding proteins are found in both cytoplasm and nucleus of tumour and other cells (Cho-Chung *et al.*, 1978), cathepsin D is a lysosomal enzyme of both tumour cells and macrophages (Henry *et al.*, 1990; O'Donoghue *et al.*, 1992; Stonelake *et al.*, 1994). Cyclic AMP-binding has also been detected in macrophages (Yamamoto *et al.*, 1987) and it may be that the inter-relationship between cathepsin D and cyclic AMP-finding relates to this. A weaker association was found between cyclic AMP-binding protein(s) and ER concentration, and this might simply reflect the fact that more cellular tumour specimens yield both more ER (when positive) and more cyclic AMP-binding protein(s). As has been shown previously in many other studies including our own, there was a significant inverse relationship between ER and EGFR.

After a median follow-up of 3 years, for risk of either any recurrence or death, ER concentration, node status and tumour grade remained the only significant *independent* factors among those examined. For risk of distant recurrence, clinical tumour size made a slight additional contribution. For risk of local recurrence, a risk generally associated with certain histopathological features (Paterson *et al.*, 1992), only tumour grade was important by multivariate stepwise analysis, although grade was not reported in that earlier study. It should be noted that in the present study, however, there were relatively few local recurrences and, therefore, the power to identify significant prognostic factors is limited. These findings are in line with those of our previous report (Hawkins *et al.*, 1991) in a different, heterogeneous group of patients ($n = 123$) of poorer prognosis, presenting at eleven different hospitals rather than at a single Breast Unit. The finding that tumour grade was of strong prognostic significance is in agreement with the results of multiple studies from other centres, particularly the Nottingham group e.g. Haybittle *et al.* (1982), plus one of our own earlier studies (Hawkins *et al.*, 1987*a*). Cytosolic protein concentration, reported previously by Soreide *et al.* (1991) to be of some prognostic value, was found to be of significance here only in a univariate analysis; its value disappeared on multivariate analysis.

In the present work, neither cathepsin D nor cyclic AMP-binding proteins were found to be of significance in influencing disease-free interval. This was unexpected in the light of previous work. High levels of cathepsin D have been shown to be associated with a poorer prognosis in most (Rochefort *et al.*, 1990), but not all (e.g. Henry *et al.*, 1990; Stonelake *et al.*, 1994), previous studies. However, two recent histochemical studies (Castiglioni *et al.*, 1994; Roger *et al.*, 1994) have confirmed the complexity of the situation, in that cathepsin D is not confined to malignant cells and Cardiff (1994), in an associated editorial, cites methodology and type of antibody used as potentially confounding factors. Johnson *et al.* (1993) demonstrated that, in cell lines, cathepsin D is not correlated with invasiveness, reinforcing the view that the prognostic role of cathepsin D probably relates to high levels of cathepsin D in stromal components such as infiltrating inflammatory cells, although Stonelake *et al.* (1994) consider that tumour aggressiveness/more advanced disease may be associated with concomitant increased expression of cathepsin D by both malignant epithelial cells and macrophages.

It may be that similar considerations apply to cyclic AMP-binding proteins, i.e. that these, too, being found in both tumour cells and macrophages (Yamamoto *et al.*, 1987), may reflect the degree of inflammation in the tumour, rendering high levels of these proteins a sign of poor prognosis. In a previous, retrospective study (Miller *et al.*, 1990), we have found that patients with high levels of cyclic AMP binding had a very short disease-free survival, this finding being confirmed in a second, prospective study (Miller *et al.*, 1993). Careful comparison of these previous studies with the present work shows some changes over the years during which the studies were conducted: 1979–84 (Miller *et al.*, 1990); 1984–87 (Miller *et al.*, 1993); and 1990–91 (present work). There have been changes in (1) the proportion of patients available for study from the whole population attending our Breast Clinic (12%, 15% and 33% respectively in the references Miller *et al.*, 1990; Miller *et al.*, 1993 and the present work); (2) the proportion of patients studied who had high levels (>8000 fmol $mg^{-1}$ protein) of cyclic AMP-binding in their tumours (12%, 9% and 3% respectively); and (3) the proportion of patients studied who received adjuvant or primary endocrine therapy (40%, 67% and 80% respectively). Thus, the most likely explanations for the differences between the present and earlier studies are that: (1) here, follow-up is relatively short (median 36.2 months); (2) the patient population (approximately 33% of those presenting to our Unit) differs from those previously examined, which tended to include patients with larger tumours, higher levels of cyclic AMP-binding proteins and poorer prognosis; (3) a large proportion (80%) of the present study group of patients received adjuvant or primary endocrine therapy, mostly tamoxifen, which may have increased the apparent importance of ER concentration in influencing outcome; (4) it should be noted that in the present study, we have analysed ER concentration as a continuous variable. Despite the recommendation of statisticians, e.g. Altman (1991), Simon *et al.* (1994) to do this, a very large number of studies continue to classify ER as a dichotomous variable (+ or −) using an arbitrary cut-off and find ER of little prognostic value. Use of an inappropriate cut-off can destroy the value of almost any prognostic factor.

The quantitative importance of ER measurements found here (quartiles <11, 11–64, 64–164 and >164 fmol $mg^{-1}$ protein) is in agreement with the views of Shek and Godolphin (1989) who, in a larger series of patients (1184), found marked differences for patients with $\leqslant 1$, 2–9, 10–159 and $\geqslant 160$ fmol $mg^{-1}$ protein. The most useful prognostic factors are likely to change with the course of the disease and, thus, follow-up time may be critical in relation to the prognostic power of a given factor; in the extreme case, for example, as Stoll (1992) has pointed out, any factors which have prognostic significance initially at presentation may have less value after relapse and the initial list of prognostic indices to be considered may well require revision at that event. In the present work, analyses at two time points (16.5 months and 36.2 months) have shown little change; only tumour size has become of significance for risk of recurrence at a distant site after 3 years. However, in the light of Stoll's comments, we propose to update our study after further follow-up.

The present study found no evidence that EGFR measurements offer prognostic information additional to that provided by ER concentration, node status and tumour grade. These findings are also in line with our previous study (Hawkins *et al.*, 1991) and the results of multivariate analyses by Dutch workers (Koenders *et al.*, 1993; Foekens *et al.*, 1989) and may perhaps relate to factors other than the four listed above. In the present study, in order to be able to include as many tumours (irrespective of size) as possible, we changed our method for EGFR assay from a multiple-dose one with Scatchard analysis (Hawkins *et al.*, 1991) to a single saturating-dose assay. Neither method, in our experience, is ideal, and concern has been expressed over the methodology of this assay (Koenders *et al.*, 1992). However, two other considerations may be of greater significance, as discussed previously (Hawkins, 1993). Firstly, there is a strong inverse relationship between ER and EGFR measurements and, where the former are satisfactory, they may enter the Cox statistical model first and eliminate a contribution from the latter. Secondly, there appears to be no clear difference between EGFR levels in benign and malignant breast tissues (compare EGFR levels in benign tissues 0–5.11 fmol $mg^{-1}$ membrane protein, $n = 18$, and malignant tissues which, with the exception of three tumours, were all in the range 0–6.43 fmol $mg^{-1}$ membrane protein, $n = 215$; see Methods section and Barker *et al.*, 1989; Dittadi *et al.*, 1993), rendering more significant the possible contribution of any benign material homogenised in the 'tumour' specimen (compare ER levels in benign tissues 2–102 fmol $mg^{-1}$ protein, $n = 18$, and malignant tissues 0–1511 fmol $mg^{-1}$ protein, $n = 2600$, unpublished observations.).

In summary, we have shown that patients with operable breast cancer are at increased risk of recurrence within 1–3 years when ER-negative and/or node-positive and/or of low tumour grade; this recurrence being most likely to be at a distant site.

It is concluded that in the early follow-up period, ER concentration is of perhaps greater prognostic significance than is generally appreciated, especially when measured with careful quality control of both assay and tumour specimen; other biochemical factors of larger/additional prognostic significance remain to be established.

**Acknowledgements**
This study was supported by grants from the University of Edinburgh Cancer Research Fund and the Melville Trust for the Care and Cure of Cancer. We wish to acknowledge also a generous donation made by the girls of Luxmore House, The King's School, Canterbury, who, as part of their Special Effort project, raised £1000 in September 1994 to support this research. The oestrogen receptor assays were performed as part of a routine service funded by the Lothian Health Board. We thank Messrs Jian'an Luan,

Ji-Xian Wang and Philip Mowbray for helping with the statistical analyses, Walter Hawkins and Dorothy Gray for histological processing of the tumour specimens and the Department of Pathology, University of Edinburgh for providing the tumour specimens for analyses and for confirming or otherwise the presence of viable tumour. We thank Marion Walker for help in collating these data and preparing the manuscript.

### References

ALTMAN DG. (1991). Categorising continuous variables. *Br. J. Cancer*, **64**, 975.

BARKER S, PANAHY C, PUDDEFOOT JR, GOODE AW AND VINSON GP. (1989). Epidermal growth factor receptor and oestrogen receptors in the non-malignant part of the cancerous breast. *Br. J. Cancer*, **60**, 673-677.

BLOOM HJG AND RICHARDSON WW. (1957). Histological grading and prognosis in breast cancer. *Br. J. Cancer*, **11**, 359-377.

BRADFORD MM. (1976). A rapid and sensitive method for the quantitation of microgram quantities of protein utilising the principle of protein-dye binding. *Anal. Biochem.*, **72**, 248-254.

BROWN JB, BULBROOK RD AND GREENWOOD FC. (1957). An evaluation of a chemical method for the estimation of oestriol, oestrone and oestradiol-17b in human urine. *J. Endocrinol.*, **16**, 41-48.

CASTIGLIONI T, MERINO MJ, ELSNER B, LAH TT, SLOANE BF AND EMMERT-BUCK MR. (1994). Immuno-histochemical analysis of cathepsins D, B and L in human breast cancer. *Hum. Pathol.*, **25**, 857-862.

CARDIFF RD. (1994). Cathepsin D and breast cancer: Useful? *Hum. Pathol.*, **25**, 847-848.

CHO-CHUNG YS, BODWIN JS AND CLAIR T. (1978). Cyclic AMP-binding proteins: inverse relationship with estrogen receptors in hormone-dependent mammary tumour regression. *Eur. J. Biochem.*, **86**, 51-60.

CREN H, LECHEVREL C, ROUSSEL G AND GOUSSARD J. (1991). Evolution of immunoreactivity of monoclonal antibodies H222 and/or D547 used in the detection of breast cancer estrogen receptors. Varying reactivity of receptor isoforms. *J. Steroid Biochem. Mol. Biol.*, **39**, 519-527.

DITTADI R, DONISI PM, BRAZZALE A, CAPPELLOZZA G, BRUSCAGNIN G AND GION M. (1993). Epidermal growth factor receptor in breast cancer. Comparison with non-malignant breast tissue. *Br. J. Cancer*, **67**, 7-9.

DIXON JM, PAGE DL, ANDERSON TJ, LEE D, ELTON RA, STEWART HJ AND FORREST APM. (1985). Long-term survivors after breast cancer. *Br. J. Surg.*, **72**, 445-448.

FISHER ER, ANDERSON S, REDMOND C AND FISHER B. (1993). Pathological findings from the national surgical adjuvant breast project Protocol B-06. *Cancer*, **71**, 2507-2514.

FOEKENS JA, PORTENGEN H, VAN PUTTEN WJL, TRAPMAN AMAC, REUBI J-C, ALEXIEVA-FIGUSH J AND KLIJN JGM. (1989). Prognostic value of receptors for insulin-like growth factor 1, somatostatin, and epidermal growth factor in human breast cancer. *Cancer Res.*, **49**, 7002-7009.

GASPARINI G, BEVILACQUA P, POZZA F, MELI S, BORACCHI P, MARUBINI E AND SAINSBURY JRC. (1992). Value of epidermal growth factor receptor status compared with growth fraction and other factors for prognosis in early breast cancer. *Br. J. Cancer*, **66**, 970-976.

HAWKINS RA. (1993). Prognostic factors: seeking a shaft of light or getting lost in the woods? A personal view from Sherwood Forest. *The Breast*, **2**, 125-129.

HAWKINS RA, BLACK R, STEELE RJC, DIXON JMJ AND FORREST APM. (1981). Oestrogen receptor concentration in primary breast cancer and axillary node metastases. *Breast Cancer Res. Treat.*, **1**, 245-251.

HAWKINS RA, WHITE G, BUNDRED NJ, DIXON JMJ, MILLER WR, STEWARD HJ AND FORREST APM. (1987*a*). Prognostic significance of oestrogen and progesterone receptor activities in breast cancer. *Br. J. Surg.*, **74**, 1009-1013.

HAWKINS RA, SANGSTER K, TESDALE AL, FERGUSON WA, KRAJEWSKI A, LEVACK PA AND FORREST APM. (1987*b*). Experience with new assays for oestrogen receptors using monoclonal antibodies. *Biochem. Soc. Trans.*, **15**, 949-950.

HAWKINS RA, KILLEN E, WHITTLE IR, JACK WJL, CHETTY U AND PRESCOTT RJ. (1991). Epidermal growth factor receptors in intracranial and breast tumours: their clinical significance. *Br. J. Cancer*, **63**, 553-560.

HAYBITTLE JL, BLAMEY RW, ELSTON CW, JOHNSTON J, DOYLE PJ, CAMPBELL FC, NICHOLSON RI AND GRIFFITHS K. (1982). A prognostic index in primary breast cancer. *Br. J. Cancer*, **45**, 361-366.

HENRY JA, McCARTHY AL, ANGUS B, WESTLEY BR, MAY FEB, NICHOLSON S, CAIRNS J, HARRIS AL AND HORNE CHW. (1990). Prognostic significance of the estrogen-regulated protein, cathepsin D in breast cancer: an immunohistochemical study. *Cancer*, **65**, 265-271.

HUMENIUK V, HAWKINS RA, PRESCOTT RJ, ROBERTS MM, STEWART HJ AND FORREST APM. (1982). Oestrogen receptors and primary breast cancer. *Aust. NZ J. Surg.*, **52**, 408-414.

JENSEN EV. (1975). Estrogen receptors in hormone-dependent breast cancers. *Cancer Res.*, **35**, 3362-3364.

JOHNSON MD, TORRI JA, LIPPMAN ME AND DICKSON RB. (1993). The role of cathepsin D in the invasiveness of human breast cancer cells. *Cancer Res.*, **53**, 873-877.

KOENDERS PG, FAVERLY D, BEEX LVAM, BRUGGINK EDM, KIENHUIS CBM AND BENRAAD ThJ. (1992). Epidermal growth factor receptors in human breast cancer: a plea for standardisation of assay methodology. *Eur. J. Cancer*, **28**, 693-697.

KOENDERS PG, BEEX LVAM, KIENHUIS CBM, KLOPPENBORG PWC AND BENRAAD ThJ. (1993). Epidermal growth factor receptor and prognosis in human breast cancer: a prospective study. *Breast Cancer Res. Treat.*, **25**, 21-27.

MILLER WR, SENBANJO RO, TELFORD J AND WATSON DMA. (1985). Cyclic AMP binding proteins in human breast cancer. *Br. J. Cancer*, **52**, 531-535.

MILLER WR, ELTON RA, DIXON JMJ, CHETTY U AND WATSON DMA. (1990). Cyclic AMP binding proteins and prognosis in breast cancer. *Br. J. Cancer*, **61**, 263-266.

MILLER WR, WATSON DMA, JACK WJL, CHETTY U AND ELTON R. (1993). Tumour cyclic AMP binding proteins: an independent prognostic factor for disease recurrence and survival in breast cancer. *Breast Cancer Res. Treat.*, **26**, 89-94.

NICHOLSON S, SAINSBURY JRC, NEEDHAM GK, CHAMBERS P, FARNDON JR AND HARRIS AL. (1988). Quantitative assays of epidermal growth factor receptor in human breast cancer: cut-off points of clinical relevance. *Int. J. Cancer*, **42**, 36-41.

O'DONOGHUE A, GALEA M, BELL JA, ELSTON CW, BLAMEY RW AND ELLIS IO. (1992). Cathepsin D reactivity in human breast carcinoma, important prognostic factor or epiphenomenon? *The Breast*, **1**, 159.

OSBORNE CK. (1992). Prognostic factors for breast cancer: have they met their promise? *J. Clin. Oncol.*, **10**, 679-682.

PATERSON DA, ANDERSON TJ, JACK WJL, KERR GR, RODGER A AND CHETTY U. (1992). Pathological features predictive of local recurrence after management by conservation of invasive breast cancer: importance of non-invasive carcinoma. *Radiother. Oncol.*, **25**, 176-180.

ROCHEFORT H. (1990). Cathepsin D in breast cancer. *Breast Cancer Res. Treat.*, **16**, 3-13.

ROGER P, MONTCOURRIER P, MAUDELONDE T, BROUILLET J-P, PAGES A, LAFFARGUE F AND ROCHEFORT H. (1994). Cathepsin D immunostaining in paraffin-embedded breast cancer cells and macrophages: correlation with cytosolic assay. *Hum. Pathol.*, **25**, 863-871.

SAEZ S, CHEIX F AND ASTELAIN B. (1983). Prognostic value of estrogen and progesterone receptors in primary breast cancer. *Breast Cancer Res. Treat.*, **3**, 345-354.

SAINSBURY JRC, FARNDON JR, NEEDHAM GK, MALCOLM AJ AND HARRIS AL. (1987). Epidermal growth factor receptor status as a predictor of early recurrence and death from breast cancer. *Lancet*, **1**, 1398-1402.

SCATCHARD G. (1949). The attraction of proteins for small molecules and ions. *Ann. NY. Acad. Sci.*, **51**, 660-672.

SIMON R AND ALTMAN DG. (1994). Statistical aspects of prognostic factor studies in oncology. *Br. J. Cancer*, **69**, 979.

SHEK LL AND GODOLPHIN W. (1989). Survival with breast cancer: the importance of oestrogen receptor quantity. *Eur. J. Clin. Oncol.*, **25**, 243-250.

SØREIDE JA, LEA OA AND KVINNSLAND S. (1991). Cytosol protein content and prognosis in operable breast cancer. Correlations with steroid hormone receptors and other prognostic factors. *Breast Cancer Res. Treat.*, **20**, 25-32.

STONELAKE PS, BAKER PG, GILLESPIE WM, DUNN JA, SPOONER D, MORRISON JM, BUNDRED NJ, OATES GD, LEE MJR, NEOPTOLEMOS JP, CHAN SY AND BAKER PR. (1994). Steroid receptors, pS2 and cathepsin D in early clinically node-negative breast cancer. *Eur. J. Cancer*, **30A**, 5-11.

STOLL BA. (1992). Prognostic indices in breast cancer. In *Pointers to Cancer Prognosis*, Stoll BA (ed.) chap. 13. Martinus Nijhoff: Dordrecht.

YAMAMOTO H AND SUZUKI T. (1987). Prostaglandin $E_2$-induced activation of adenosine 3'5' cyclic monophosphate-dependent protein kinases of a murine macrophage-like cell line (P388D1). *J. Immunol.*, **139**, 3416-3421.